# Spatially-Resolved Influence of Temperature and Salinity on Stock and Recruitment Variability of Commercially Important Fishes in the North Sea

**DOI:** 10.1371/journal.pone.0161917

**Published:** 2016-09-01

**Authors:** Anna Akimova, Ismael Núñez-Riboni, Alexander Kempf, Marc H. Taylor

**Affiliations:** Thünen Institute of Sea Fisheries, Hamburg, Germany; Havforskningsinstituttet, NORWAY

## Abstract

Understanding of the processes affecting recruitment of commercially important fish species is one of the major challenges in fisheries science. Towards this aim, we investigated the relation between North Sea hydrography (temperature and salinity) and fish stock variables (recruitment, spawning stock biomass and pre-recruitment survival index) for 9 commercially important fishes using spatially-resolved cross-correlation analysis. We used high-resolution (0.2° × 0.2°) hydrographic data fields matching the maximal temporal extent of the fish population assessments (1948–2013). Our approach allowed for the identification of regions in the North Sea where environmental variables seem to be more influential on the fish stocks, as well as the regions of a lesser or nil influence. Our results confirmed previously demonstrated negative correlations between temperature and recruitment of cod and plaice and identified regions of the strongest correlations (German Bight for plaice and north-western North Sea for cod). We also revealed a positive correlation between herring spawning stock biomass and temperature in the Orkney-Shetland area, as well as a negative correlation between sole pre-recruitment survival index and temperature in the German Bight. A strong positive correlation between sprat stock variables and salinity in the central North Sea was also found. To our knowledge the results concerning correlations between North Sea hydrography and stocks’ dynamics of herring, sole and sprat are novel. The new information about spatial distribution of the correlation provides an additional help to identify mechanisms underlying these correlations. As an illustration of the utility of these results for fishery management, an example is provided that incorporates the identified environmental covariates in stock-recruitment models.

## Introduction

Environmental conditions and fisheries are main drivers of variability of marine ecosystems [1], [2], [3]. Profound responses of marine ecosystems to their changing environment have been reported all over the world at various spatial and temporal scales and trophic levels [4], [3], [5]. Environmental changes are believed to trigger observed changes in abundances, composition of marine organisms and ecosystem regime shifts [6], [7], [8], [9]. Commercially exploited fishes are an important component of fish communities in many ecosystems. Therefore our understanding of the variability of these fishes and its driving factors is not only important for optimization of the fishery management, but may also assist in predicting the effects of climate variability on whole ecosystem.

The North Sea is a complex shelf sea ecosystem, which has undergone strong changes over the last decades, mainly in zooplankton and fish communities [10], [11], [12]. These changes are believed to be primarily driven by environmental variability and heavy exploitation of the commercial fishes [13], [14], [15]. The North Sea fisheries target more than twelve commercially important species, in addition to a substantial amount of by-catch. Gadoid species, like cod (*Gadus morhua*), whiting (*Merlangius merlangus*), haddock (*Melanogrammus aeglefinus*) and saithe (*Pollachius virens*), as well as flatfish species, like plaice (*Pleuronectes platessa*) and sole (*Solea solea*), are targeted by mixed demersal fisheries. The pelagic fishery mainly targets herring (*Clupea harengus*) but also includes mackerel (*Scomber scombrus*) and horse mackerel (*Trachurus trachurus*). In addition, there is an industrial fishery for sandeel (*Ammodytes spec*.), sprat (*Sprattus sprattus*) and Norway pout (*Trisopterus esmarkii*).

Commercial stocks in the North Sea are well documented and show a high inter-annual variability in their biomass and productivity [13], [15]. Together with fishing pressure this led to collapses and recoveries of the North Sea stocks, for instance, herring (e.g. [16]) and cod (e.g. [17]). The reasons of the variability and collapses of many stocks are still debated and are likely to involve different mechanisms for each species.

Correlation and regression analyses have been widely used to study relations between environmental factors and stock parameters of the North Sea fish stocks (e.g. [18], [19], [17], [20], [21], [22]). Previous studies dealt mainly with the influence of abiotic factors on recruitment and used various sources of information about environmental conditions in the North Sea. Some studies have used large-scale oceanic or atmospheric indexes as a proxy for local North Sea hydrography, e.g. the North Atlantic Oscillation Index (NAO; [23], [21]) or the Atlantic Multidecadal Oscillation Index (AMO; [8], [24], [25]). These studies helped to explain similarities of changes in different stocks all over the North-Atlantic by attributing them to large-scale oceanic processes. This approach might have a serious disadvantage when the link between the indices and local environmental conditions at various lags is weak [26]. Furthermore, such relationships are difficult to interpret, because large-scale circulation patterns normally have a complex footprint on the local conditions and an exact mechanism behind such correlations might remain unresolved or misinterpreted.

Another group of studies exploited observations either at a particular location [27], [28], [29], [30] or used various gridded products of sea surface temperature (SST; [19], [20], [24], [31]). An advantage of using SST or *in situ* observations was that local temperature conditions could be addressed. But the coarse resolution of available data did not allow for analyses of spatially-resolved correlations [19], [32]. In the majority of previous studies SST data was averaged over the entire North Sea to provide a single time series for the analyses.

Recently an increasing number of studies addresses spatial heterogeneity of the North Sea and demonstrates the importance of spatial scales in the interactions between hydrography and fishes [33], [34]. In this study we continue in this direction by investigating the spatially-resolved influence of temperature and salinity on nine commercially important fishes in the North Sea using stock assessment data and a new reconstruction of the North Sea hydrography over the past six decades [35]. A high spatial and vertical resolution of the North Sea hydrography allowed for the identification of regions and depths with the strongest correlation between environmental and stock variables. A precise identification of such regions may help to identify key processes driving the variability of the fish biomass and productivity and increase the explanatory power of the environmental variables used in stock-recruitment models, as we show below.

## Data

**Fig 1:**
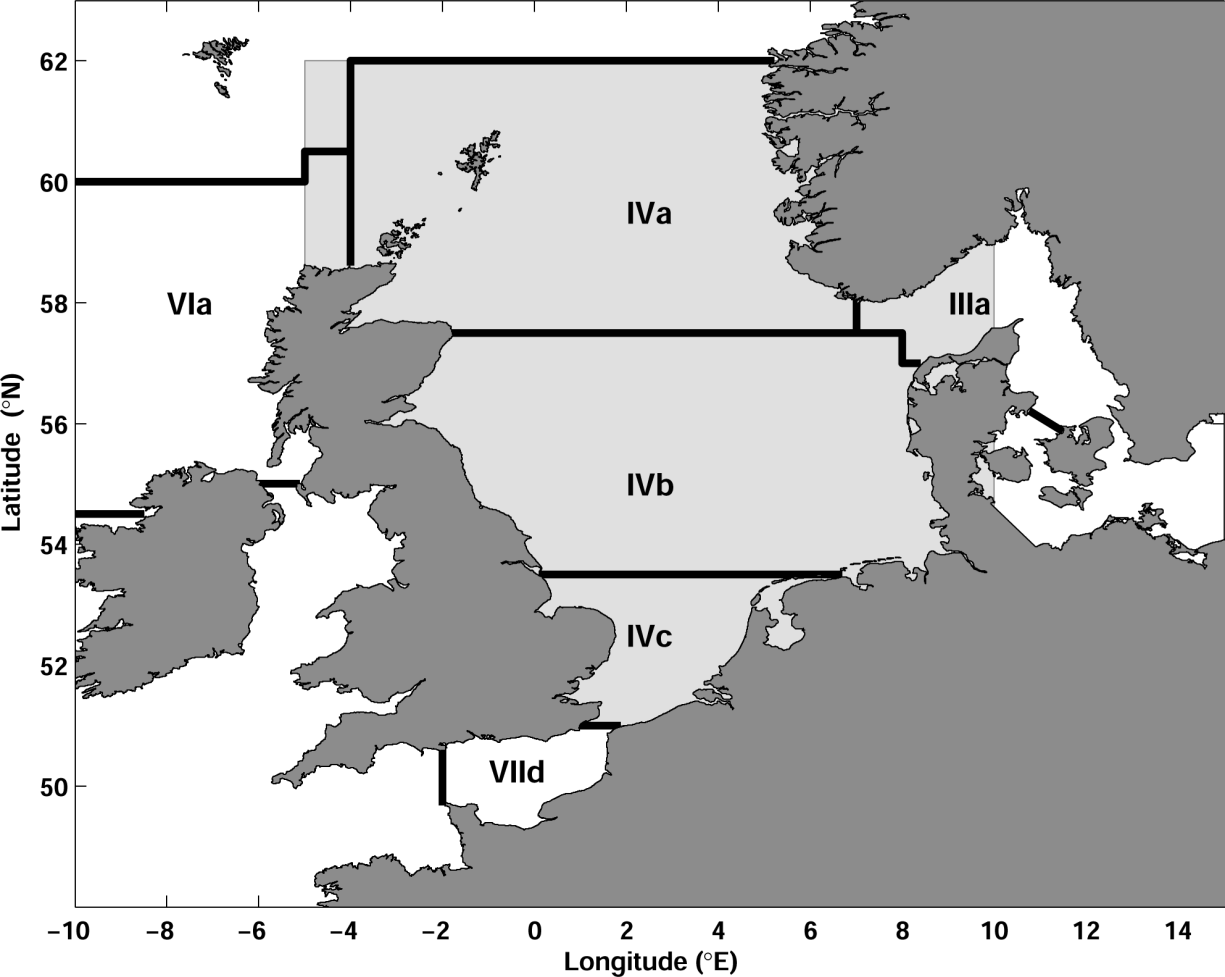
Study area in the North Sea. The domain of AHOI (Adjusted Hydrography Optimal Interpolation) is shown in light gray. Polygons show ICES (International Council for the Exploration the Sea) stock areas for the stock assessment units used in this study (see Table 1).

To study the relationships between fish stocks and environmental conditions we used a recent reconstruction of the North Sea hydrography [35]. This Adjusted Hydrography Optimal Interpolation (AHOI) represents monthly maps of temperature and salinity, obtained for 54 depths in the North Sea for the period from 1948 to 2013 with a horizontal resolution of 0.2°×0.2°. The data covers the whole North Sea from 50°N to 62°N and from 5°W to 10°E (Fig 1). Within the scope of this study, AHOI was further developed and improvements in comparison to [35] are described in Section ‘The North Sea hydrography’. We used here AHOI version 8.11, which is available via Figshare data portal [36].

Recruitment (R), spawning stock biomass (SSB) and fishing mortality (F) time series of 9 assessed fish stocks in the North Sea (Table 1, Fig 1) were obtained from the database of the International Council for the Exploration the Sea (ICES; [37]). We used main commercially important stocks with analytical assessments (ICES category 1 assessments) with the exception of plaice and sole in Subarea VIId, because the AHOI data does not include the English Channel (Fig 1). We used the assessments conducted by ICES working groups in 2015 using information from catch statistics and scientific surveys [13], [15]. Assessments were not downscaled if they included areas outside the North Sea (e.g., VIa or VIId in Fig 1) under the assumption that the main part of the stocks is distributed in ICES area IV (Fig 1). SSB is given in tons, whereas R is provided as the number of individuals and usually refers to the youngest age of the fishes caught in the fisheries or caught quantitatively in scientific surveys. The extent of the time series used in this study, as well as stock specific age of recruitment are summarized in Table 1. Since the AHOI dataset covers the period from 1948 to 2013 we were able to include the complete time series of stock assessment for all stocks, except herring, for which the first year of the time series (1947) was excluded from the analysis.

**Table 1:** Fish species and their stock areas used in this study. Age at recruitment (RA) and analyzed time periods are shown.

Species	Stock area	RA	Time period
Atlantic cod (*Gadus morhua*)	Subarea IV, Divisions VIId and IIIa	1	1963–2014
Atlantic herring (*Clupea harengus*)	Subarea IV, Divisions IIIa and VIId	0	1948–2013
European sprat (*Sprattus sprattus*)	Subarea IV	0	1974–2013
Europen plaice (*Pleuronectes platessa*)	Subarea IV	1	1957–2014
haddock (*Melanogrammus aeglefinus*)	Subarea IV, Divisions IIIa and VIa	0	1972–2013
Norway Pout (*Trisopterus esmarkii*)	Subarea IV and IIIa	0	1983–2013
whiting (*Merlangius merlangus*)	Subarea IV, Division VIId	1	1990–2014
Common sole (*Solea Solea*)	Subarea IV	1	1957–2014
saithe (*Pollachius virens*)	Subarea IV and VI, Division IIIa	3	1967–2014

## Methods

### The North Sea Hydrography

AHOI is based on Gauss-Markov interpolation of *in situ* observations of temperature and salinity in the North Sea [35]. The results of the interpolation were adjusted with harmonic analysis and with a routine for vertical density stability of the World Ocean Atlas [38]. The method has been described and validated in Núñez-Riboni and Akimova [35] (from now on: NRA15), where further details can be found. In comparison to NRA15, we introduced the following improvements to the North Sea AHOI dataset.

1. The time series excluded from the analysis to validate AHOI in NRA15 were included in the interpolation run used in this study (see Table 2 of NRA15).
2. In NRA15, bounds for upper and lower hydrography outliers were calculated by averaging the largest and the smallest observations inside overlapping neighborhoods around the grid points. In the present model run, these bounds were calculated by fitting a “running” cubic surface. This should better represent the outlier’s bounds near strong gradients of the mean field.
3. The adjustment for vertical stability, which was applied only to individual monthly hydrography maps in NRA15, was applied to the mean fields as well.

**Table 2:** Results of the field significance test for stock (*B*) and hydrographical variables (*H*), which revealed significant correlations at time lag τ.

Species	*B*	*H*	τ	*M*_*0*_ (5m)	*M*_*0*_ (bottom)	Field significance test
cod	R_c_	T_JFM_	1	2.97	6.94	passed
cod	R_c_/SSB_c_	T_JFM_	0	3.08	8.22	passed
herring	SSB_h_	T_JFM_	0	3.53	4.76	passed
herring	R_h_/SSB_h_	T_JFM_	0	3.44	5.81	passed
sprat	R_sp_	S_OND_	0	9.13	8.80	passed
sprat	SSB_sp_	S_OND_	0	11.26	9.71	passed
plaice	R_p_	T_AMJ_	1	1.02	8.73	passed
plaice	R_p_/SSB_p_	T_AMJ_	0	3.17	4.89	passed
haddock	SSB_ha_	T_JAS_	0	5.48	11.81	failed
haddock	R_ha_	S_AMJ_	1	13.73	9.51	failed
Norway pout	R_np_	S_AMJ_	1	8.93	13.27	failed
Norway pout	R_np_/SSB_np_	S_JFM_	0	13.40	11.28	failed
sole	R_s_	T_AMJ_	2	2.52	7.59	passed
sole	R_s_/SSB_s_	T_AMJ_	1	7.01	11.01	passed
saithe	SSB_sa_	T_OND_	4	7.41	9.45	failed
saithe	R_sa_	S_AMJ_	3	8.89	9.94	failed

The number of grid points *M*_*0*_ (in % of the total grid points) obtained with hydrographical variables at depth of 5 m and at the bottom are shown. The subscripts of the stock variables refer to corresponding fish species (c–cod, h–herring, sp–sprat, p–plaice, ha–haddock, np–Norway pout, s–sole, sa–saithe). The subscripts of the hydrological variables refer to the season of the strongest correlation (JFM—January to March, AMJ—April to June, JAS—July to September and OND—October to December).

### Correlation Analysis

We explored relationships between environmental conditions and stock variables by means of spatially-resolved cross-correlation analysis. We calculated the Pearson cross-correlation between stock and hydrographical time series for each grid point of AHOI at various time lags τ [39]:

$$Ρ(\tau )=\frac{1}{\sigma_{b}\sigma_{h}}\frac{1}{N-|\tau |}\sum_{i=1}^{N-\tau }h_{i+\tau }(x,y,z)b_{i},$$ (1)

where *b*_*i*_ are anomalies of a stock variable *B*, *h*_*i*+*τ*_(*x*,*y*,*z*) are anomalies of a hydrographical variable *H* at time lag *τ* and at grid point (*x*, *y*, *z*), with latitude *x* and longitude *y* and depth *z*. *σ*_*b*_ and *σ*_*h*_ are standard deviations of the time series *B* and *H*, correspondingly, and *N* is the length of the shortest time series. The anomalies *b*_*i*_ and *h*_*i*_*(x*, *y*, *z)* were obtained after low-pass filtering of the original time series with a 3-year running mean and removing linear trends within the common time period. Only cross-correlations with lags *τ* less than 20% of *N* were considered, following recommendations of Emery and Thomson [39].

We conducted cross-correlation analyses for 4 stock variables *B*: recruitment R, log-transformed recruitment ln(R), pre-recruitment survival index R/SSB and spawning stock biomass SSB. Log-transformation of R was used to linearize possible exponential relations between recruitment and environmental conditions, which have been previously reported for fish stocks in the North Sea [19] and elsewhere [40]. Pre-recruitment survival index R/SSB represents the amount of recruits per unit of SSB and is often used in recruitment-related studies [41], [42], [43]. To calculate it, R time series were shifted relative to SSB time series by the age at recruitment (Table 1).

We used temperature and salinity of AHOI as hydrographical variable *H*. Marine fishes have complex life cycles (eggs, pelagic larvae, demersal or pelagic juveniles) and different life stages may respond to changes in their environment in different ways [44]. In order to understand which season is the most critical for the recruitment success, we tested *H* averaged over various seasons: January-March (JFM), April to June (AMJ), July to September (JAS) and October to December (OND). Yearly mean hydrography JD was tested as well.

### Significance tests

The significance of obtained correlations was tested in two steps. First, we calculated confidence intervals for the cross-correlations, obtained with Eq 1, by converting P(*τ*) into a normally distributed variable z(*τ*) with the Fisher transformation [39]:

$$z(\tau )=\frac{1}{2}(\ln\left(1+Ρ(\tau \right))-ln(1-Ρ(\tau ))),$$ (2)

which has the standard error:

$$\delta_{z}=\frac{1}{({N^{*})}^{1/2}}.$$ (3)

N* is the “effective number of degrees of freedom” given by Pyper and Peterman [45]:

$$\frac{1}{N^{*}}=\frac{1}{N}+\frac{2}{N}\sum_{\tau =1}^{N}\frac{\left(N-\tau \right)}{N}\rho_{h}(\tau )\rho_{b}(\tau ),$$ (4)

where ρ_h_(τ) and ρ_b_(τ) are autocorrelations at lag *τ* of variables *h* and *b* correspondingly. Using N* instead of the length of the time series *N* in Eq 3 accounts for the autocorrelation of the time series in the calculation of the confidence limits of the cross-correlation P(*τ*) [45], [46], [20], [42]. The confidence intervals of the Fisher-transformed variable z(*τ*) were then estimated as:

$$z(\tau )-z_{p/2}\delta_{z}<z(\tau )<z(\tau )+z_{p/2}\delta_{z},$$ (5)

where *z*_*p*/2_ represents the percentiles at *p/2* of the normal distribution with zero mean and standard deviation 1. We chose p = 0.05, which corresponds to 95% confidence interval. Finally, confidence intervals for z(*τ*) were transformed back with the inverse Fisher transformation to obtain the corresponding confidence limits for *P*(*τ*) [39]. Improving over previous studies [46], [21], [47], we additionally examined the confidence of the cross-correlations at negative time lags (τ < 0) to verify that the chosen confidence limits effectively eliminated spurious correlations, i.e. correlations where *B* leads *H*.

The second significance test applied here comprised field significance. The systematical search for correlations with Eq 1 between stock variable *B* and a large amount of hydrographical time series *H* can inflate the chance of finding a significant correlation. If one of the hydrographic time series is significantly correlated with *B* 'by chance', the neighboring time series are likely to be significantly correlated with *B* as well, because the hydrographical time series are spatially-correlated. Field significance tests allow determining the minimum number of *H* time series significantly correlated with *B* that is consistent with the spatial autocorrelation of the hydrography field. To perform field significance test we applied the approach of Livezey and Chen [32], which is based on Monte-Carlo simulations. This method has been widely used in previous studies dealing with spatiotemporal correlations (e.g. [48], [49], [50], [51]). We chose here confidence level of 95% to match the significance level of the correlation analyses. The field significance test with this confidence level provides a number of the hydrographical time series M_0_ that should be equaled or exceeded in our correlation analysis such that the probability of the result occurring by chance is less than 5%. For each simulation we replaced *B* with a Gaussian-noise time series with the mean and variance identical to those of *B*. This simulated time series were then correlated with the hydrographical time series. In each simulation (400 in total) the percentage *M* of the coefficients statistically significant with 95% confidence according to Eq 1 was calculated. *M*_*0*_ was estimated as 95^th^ percentile from the histogram of *M* and was used as a threshold for the field significance. Correlations found to be significant in the test of confidence intervals (Eq 5), were rejected if the field significance test failed. For the fish species, where statistically significant correlation was found between environmental variables and SSB, we additionally tested cross-correlation between SSB and fishing mortality F using Eq 1.

## Results

Stock and environmental variables, which revealed significant correlations with 95% confidence and showed no spurious correlations at negative lags, are listed in Table 2. For these variables field significance tests were conducted. In the following sections, we present the results only for the cross-correlations, which passed both significance tests (Table 2).

### Atlantic Cod and Temperature

Three variables, namely cod recruitment R_c_, log-transformed recruitment ln(R_c_) and pre-recruitment survival index R_c_/SSB_c_ showed significant negative correlations with temperature. R_c_ and ln(R_c_) were correlated with temperature at lag 1, and R_c_/SSB_c_ at lag 0 (Table 2). All three stock variables were highly correlated to each other with the coefficients between 0.74 and 0.92, therefore they showed a similar correlation with temperature. We present here the results only for ln(R_c_) as being most pronounced.

**Fig 2:**
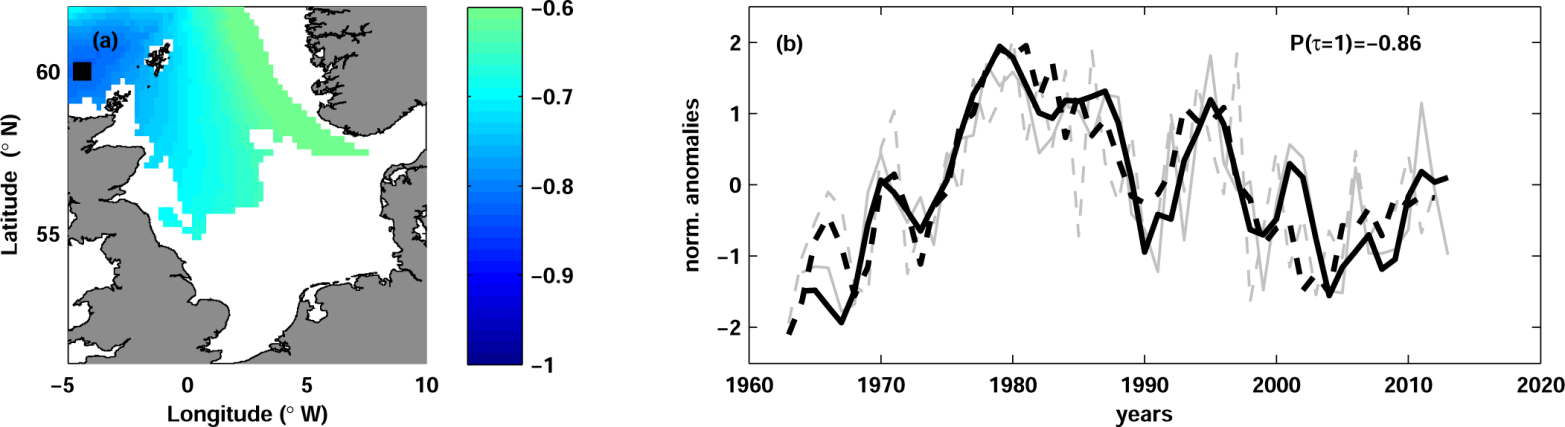
Cod log-transformed recruitment and temperature. (a) Map of correlation between log-transformed cod recruitment ln(R_c_) and temperature in JFM at lag 1 at the depth of maximal correlation (75 m). Only significant correlation is shown. The black square (60.0°N, 4.4°W) depicts the position of the maximal correlation. (b) Time series of normalized anomalies of ln(R_c_) (dashed curves) and of water temperature (solid curves) in JFM at 75 m depth and at the grid point shown with the black square in panel (a). The grey thin curves show the unfiltered time series and the thick black curves are the time series low-passed with 3-year running mean. The temperature time series are multiplied by -1 and lagged by 1 year in order to clearly show the correlation between the time series. The correlation coefficient Ρ(τ = 1) is shown.

Maps of correlation coefficients revealed significant correlation between ln(R_c_) and temperature only in the north-western North Sea (Fig 2A). Regions of the significant correlation were larger at mid-depth (40–80 m), in comparison with the upper layer, where significant correlations were obtained only north-east off the Scottish coast. The correlation was significant in all quarters, but the strongest correlation was obtained with temperature in JFM. Vertically mean significant correlation in JFM was -0.63, whereas the highest absolute correlation of -0.86 was found with temperature at 75 m depth in the north-western North Sea (Fig 2). The correlation between temperature and ln(R_c_) was strong for the whole period of observations (Fig 2B).

### Atlantic Herring and Temperature

We found a significant correlation between temperature at lag 0 and two stock variables of herring: negative correlation with pre-recruitment survival index R_h_/SSB_h_ and positive correlation with the spawning stock biomass SSB_h_. The correlation between SSB_h_ and R_h_/SSB_h_ was -0.65 and significant, whereas the correlation between R_h_ and R_h_/SSB_h_ was weaker (0.12) and insignificant. Therefore the negative correlation between R_h_/SSB_h_ and temperature seems to be a consequence of the positive correlation between temperature and SSB_h_. We describe here only the results for SSB_h_.

**Fig 3:**
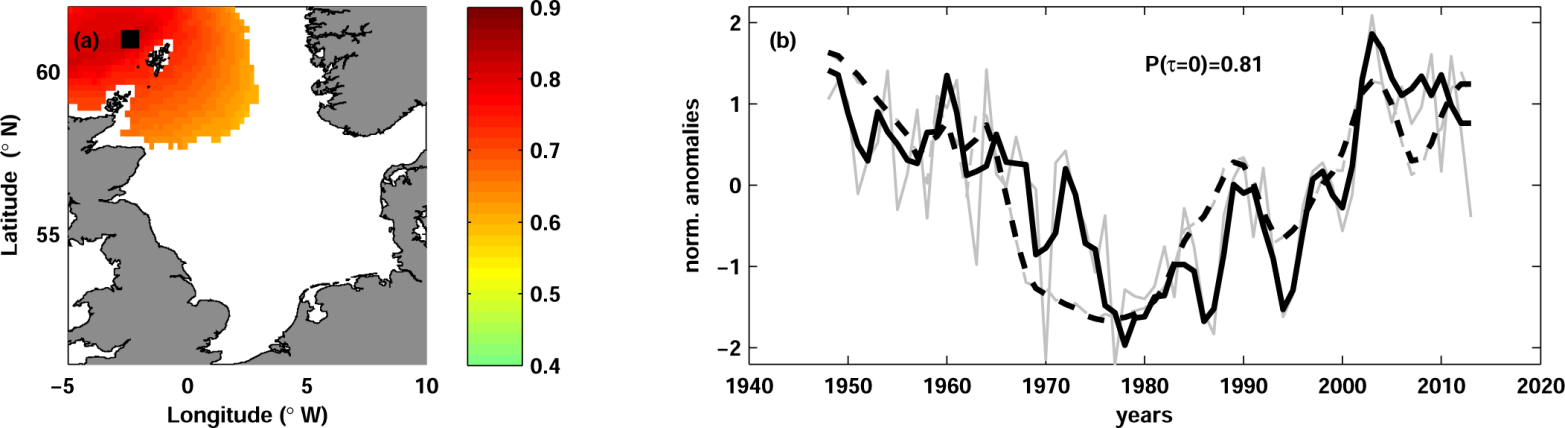
Herring spawning stock biomass and temperature. (a) Map of correlation between herring spawning stock biomass SSB_h_ and water temperature in JFM at lag 0 and at the depth of maximal correlation (60 m). Only significant correlation is shown. The black square (61.0°N, 2.4°W) depicts the position of the maximal correlation. (b) Time series of normalized anomalies of SSB_h_ (dashed curves) and water temperature (solid curves) in JFM at 60 m depth and at the grid point shown with the black square in panel (a). The grey thin curves show the unfiltered time series and the thick black curves are the time series low-passed with 3-year running mean. The correlation coefficient Ρ(τ = 0) is shown.

The correlation maps showed a region of significant correlation in the north-western North Sea (Fig 3A). Equally strong correlations were obtained in all quarters, albeit their geographical extension varied between quarters. Only in JAS the correlation became weaker and therefore partially insignificant in the upper water layers; in other quarters the correlation was significant at all depths from the surface to the bottom. The vertically mean correlation was 0.67. The highest correlation of 0.81 was observed north-west of the Shetland Islands at 60 m depth (Fig 3).

Correlation between SSB_h_ and herring fishing mortality F_h_ was found significant at lags τ = -2, -1, 0. The strongest correlation of -0.72 was found at lag -2 (fishing mortality leads SSB_h_ with a lag of 2 years).

### European Plaice and Temperature

Stock variables of European plaice revealed negative correlation with temperature in the North Sea. Significant correlations were found with plaice recruitment R_p_ at lag 1 and pre-recruitment survival index R_p_/SSB_p_ at lag 0 (Table 2). Both stock variables are highly correlated with each other (P(τ = 1) = 0.88) and hence show similar relations with temperature. The correlation with temperature was stronger for R_p_ than for R_p_/SSB_p_, therefore we use R_p_ to illustrate our results.

**Fig 4:**
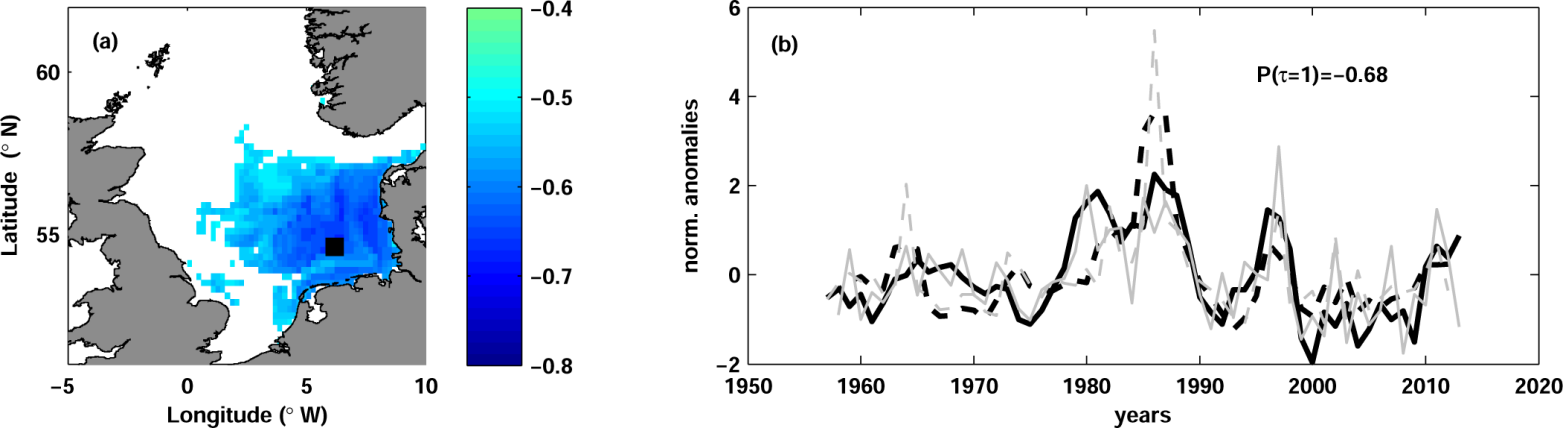
Plaice recruitment and temperature. (a) Map of correlation between plaice recruitment R_p_ and temperature in AMJ at lag 1 and at the depth of the maximal correlation (seabed). Only significant correlation is shown. The black square (54.8°N, 5.6°E) depicts the position of the maximal correlation. (b) Time series of normalized anomalies of R_p_ (dashed curves) and of temperature (solid curves) in AMJ at the seabed and at the grid point shown with the black square in panel (a). The grey thin curves show the unfiltered time series and the thick black curves are the time series low-passed with 3-year running mean. The temperature time series are multiplied by -1 and lagged by 1 year in order to clearly show the correlation between the time series. The correlation coefficient Ρ(τ = 1) is shown.

The correlation between R_p_ and temperature at lag 1 was significant in the south-eastern North Sea with maximum in the German Bight (Fig 4A). The correlation was similar at all water depths, but slightly more pronounced at the bottom. The significant correlation was found in all quarters, except NOD, albeit its spatial extent varied slightly between quarters. The strongest correlation was found with temperature in AMJ with the vertically mean of -0.57, although it was very close to the correlation with temperature in JFM (-0.53). The highest absolute correlation value of -0.68 was found at the bottom in the German Bight (Fig 4A). The correlation between R_p_ and temperature was strong from the end of 1970s onward, whereas in the beginning of the time series the correlation was rather weak (Fig 4B).

### Common Sole and Temperature

We found negative correlations between temperature and two sole variables: sole recruitment R_s_ at lag 2 and sole pre-recruitment survival index R_s_/SSB_s_ at lag 1 (Table 2). Both stock variables are highly correlated when the corresponding lags are taken into account (P(τ = 1) = 0.86). Since the correlation with R_s_/SSB_s_ was stronger than the correlation with R_s,_ we use the first variable to describe our findings.

**Fig 5:**
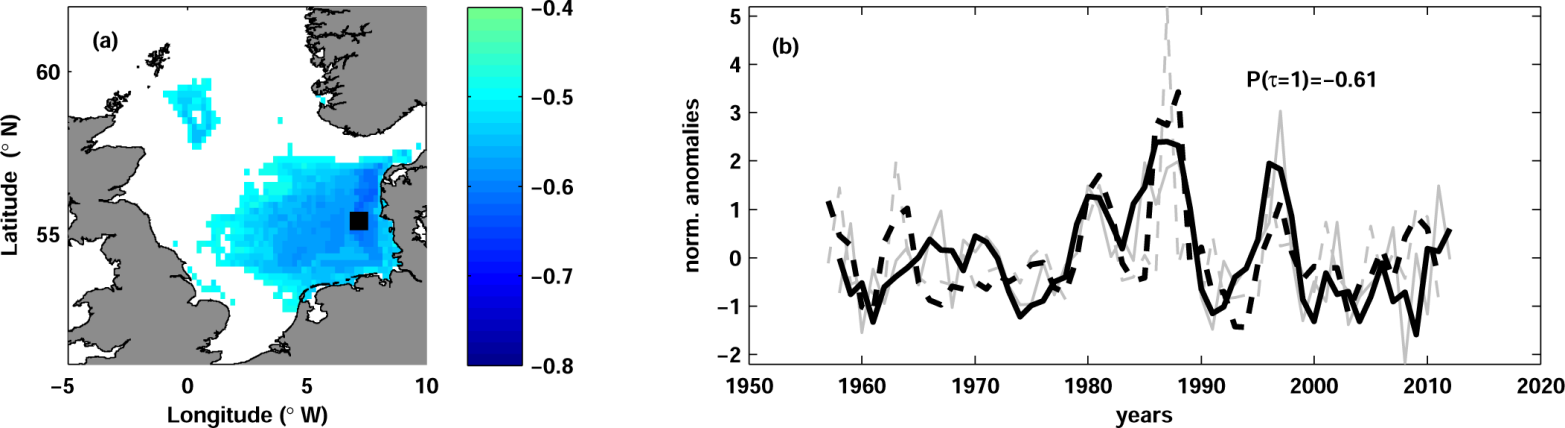
Sole pre-recruitment survival index and temperature. (a) Map of correlation between sole pre-recruitment survival index R_s_/SSB_s_ and temperature in AMJ at lag 1 and at the depth of the maximal correlation (seabed). Only significant correlation is shown. The black square (55.4°N, 7.2°E) depicts the position of the maximal correlation. (b) Time series of normalized anomalies of R_s_/SSB_s_ (dashed curves) and of temperature (solid curves) in AMJ at the seabed and at the grid point shown with the black square in panel (a). The grey thin curves show the unfiltered time series and the thick black curves are the time series low-passed with 3-year running mean. The temperature time series are multiplied by -1 and lagged by 1 year in order to clearly show the correlation between the time series. The correlation coefficient Ρ(τ = 1) is shown.

Similar to plaice, a region of significant negative correlation was found in the southern North Sea between 5 m depth and the bottom with the vertically mean correlation of -0.52. The strongest correlation of -0.61 was found at the bottom in the German Bight (Fig 5). The significant correlation was evident only in AMJ and JAS.

### European Sprat and Salinity

**Fig 6:**
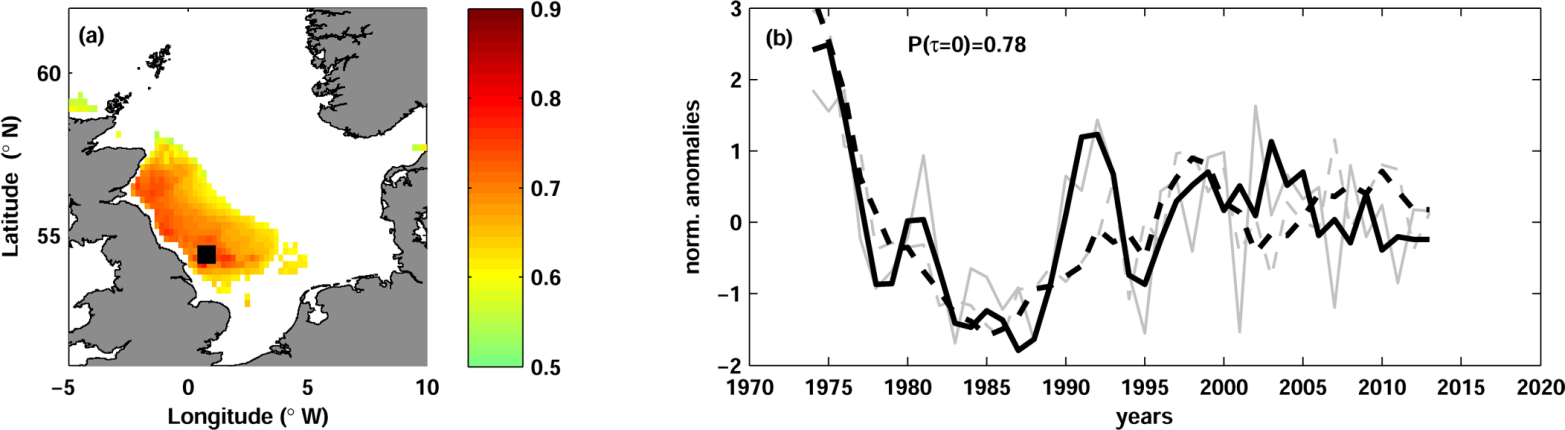
Sprat spawning stock biomass and salinity. (a) Map of correlation between sprat spawning stock biomass SSB_sp_ and salinity in OND at lag 0 and at the depth of the maximal correlation (20 m). Only significant correlation is shown. The black square (54.4°N, 0.8°W) depicts the position of the maximal correlation. (b) Time series of normalized anomalies of SSB_sp_ (dashed curves) and salinity (solid curves) in OND at 20 m depth and at the grid point shown with the black square in panel (a). The grey thin curves show the unfiltered time series and the thick black curves are the time series low-passed with 3-year running mean. The correlation coefficient Ρ(τ = 0) is shown.

**Fig 7:**
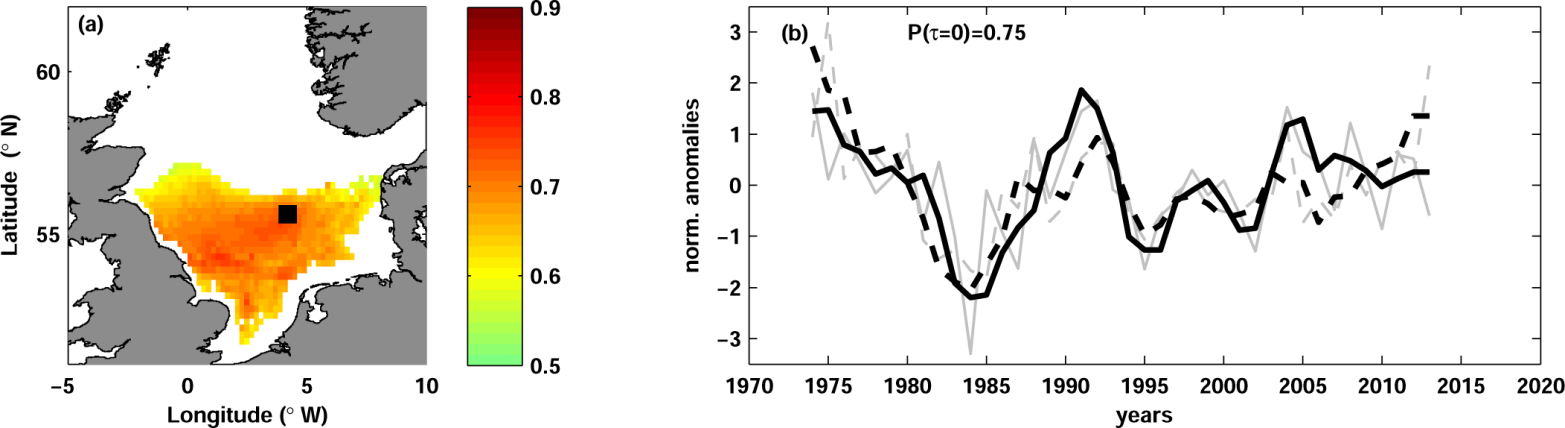
Sprat recruitment and salinity. (a) Map of correlation between sprat recruitment R_sp_ and salinity in OND at lag 0 and at the depth of the maximal correlation (10 m). Only significant correlation is shown. The black square (55.6°N, 4.2°E) depicts the position of the maximal correlation. (b) Time series of normalized anomalies of R_sp_ (dashed curves) and salinity (solid curves) in OND at 20 m depth and at the grid point shown with the black square in panel (a). The grey thin curves show the unfiltered time series and the thick black curves are the time series low-passed with 3-year running mean. The correlation coefficient Ρ(τ = 0) is shown.

The time series of sprat recruitment R_sp_ and spawning stock biomass SSB_sp_ were found to be positively correlated with salinity at lag 0 (Table 2). The correlation maps of SSB_sp_ and salinity showed significant correlations in the western North Sea along the British coast and over the Dogger Bank (Fig 6A), whereas the region of the significant correlation with R_sp_ comprises also central and southern North Sea (Fig 7A). The maximal correlation was found with salinity in the upper water layer (at 20 m depth for SSB_sp_ and at 10 m depth for R_sp_)_._ The correlation for both stock variables was most pronounced in OND, although significant correlations were found with salinity in other quarters as well, except JAS in the case of SSB_sp_ and JFM in the case of R_sp_. Vertically mean correlation with salinity in OND were 0.64 for SSB_sp_ and 0.62 for R_sp_. AHOI time series of salinity showing the strongest correlation with sprat SSB_sp_ and R_sp_ are shown in Figs 6B and 7B, correspondingly.

We tested also the correlation between SSB_sp_ and sprat fishing mortality F_sp._ We found a weak and insignificant correlation between these two variables at lag 0 (P(τ = 0) = 0.36). Correlations at other lags were also insignificant.

## Discussion

Our study aims at improving our understanding of the linkage between climatic factors and fish stocks in the North Sea by spatially explicit cross-correlation analysis. North Sea AHOI enabled us to analyze the longest time series of stock variables so far. As mentioned in the introduction, the majority of the previous studies used mainly large scale atmospheric and temperature indices [21], [25], [23], temperature data collected parallel to the fisheries data [28], [18] or SST averages over large areas of the North Sea [19], [20], [24]. All these studies missed the spatial component in their analyses. To address this gap, we studied spatially-resolved correlations between fish stocks and environmental variables in the North Sea. The goal of our study was to identify regions of the North Sea where environmental conditions seem to be more influential on stocks’ variability, as well as those with little to no influence. In the previous section we showed the most relevant regions (i.e. regions of statistically significant correlation) for five fish species. To illustrate our findings we used correlation maps for depths of maximum correlation (Figs 2–7). However, the exact depths of maximal correlations should be interpreted cautiously. Our ability to resolve spatial correlation between stock and environmental variables in the North Sea does not only depend on the spatial resolution of the available data, but is also restricted by the nature of the North Sea hydrography, i.e. by the scales of spatial (horizontal and vertical) autocorrelation of hydrographic variables. For example, temperature in the southern North Sea is highly autocorrelated vertically on the inter-annual time scales (e.g. [52]). Therefore obtained correlations between temperature in the German Bight and plaice recruitment are similar at all depths (maximal correlations at each depth are between -0.68 and -0.60) and are well within the correlation confidence limits. Using only correlative analyses, we cannot ascribe correlations obtained in this shallow region of the North Sea to the processes either taking place at the sea bottom or near the surface. The same is true for the European sole, e.g. the depth of maximal correlation cannot be regarded as a hint to a possible driving mechanism.

In contrast, the distribution of temperature in the northern extent of the North Sea is not vertically homogeneous. A seasonal thermocline at approximately 30m depth separates two water layers there (e.g. [52]). Temperature variability in these layers is driven by different mechanisms; the near-surface layer is driven mainly by the variability of the atmospheric heat fluxes, while the layer below the thermocline is driven by the temperature and strength of Atlantic Water inflow. The depth of maximal correlation obtained between herring or cod and temperature in the north-western North Sea should not be considered as exact value, but as a rough indicator of the role that near-surface or mid-depth effects might have. This will be discussed in the cases of cod and herring in the following sections.

We performed our analyses with two environmental variables (temperature and salinity) averaged quarterly without a restriction to a certain period of the year. In other words, we studied the relations between stock and hydrographic variables without presumption of a certain life-stage being the most important for the recruitment variability. This approach allowed for the identification of periods of the year having the strongest impact on the inter-annual variability of the stocks and their recruitment.

### Atlantic Cod

A negative correlation between cod recruitment and temperature has been reported previously based on the analyses of spatially averaged SST and recruitment time series (e.g., [19], [17], [53]). Various hypothesis on underlying mechanism have been proposed: match-mismatch between first-feeding larvae and zooplankton production [54], temperature related changes in zooplankton community and its productivity [55], [34] and direct effects of temperature on growth and survival of cod eggs and larvae [56], [57]. Beside bottom-up controls, top-down processes have been proposed, e.g. through changes in abundance of key predators [58] or in the predator-prey spatial overlap [59], [60].

Our study confirms previously reported strong negative correlation between cod recruitment and temperature in JFM (Fig 2). The strongest correlation was found in the north-western part of the North Sea. These results do not support the findings of Daewel et al. [54], who showed the importance of temperature in the southern North Sea for the overall recruitment success of the North Sea cod. Our analysis shows that only about 20% of the recruitment variance can be explained by the temperature variability in the southern North Sea (not shown). This compares well with almost 20% of explained variance reported in the study of Nicolas et al. [34], although direct comparison is difficult because the authors analyzed spatial and temporal variability together. In contrast to Nicolas et al. [34], the correlations with temperature in the southern North Sea found here were non-significant with our chosen significance level.

The north-western North Sea, where we found the strongest correlation, is one of the regions where Atlantic water enters the North Sea [52]. The temperature there is mainly affected by local atmospheric conditions in the combination with remote temperature changes in the North Atlantic [61]. The maximum correlation of cod recruits with temperature was found at the mid-depth (60 m) and in the region coinciding with the known distribution of Atlantic Water in the North Sea (e.g. [52]). Because the deeper waters are isolated from the atmospheric heat fluxes and mainly influenced by changes in temperature produced by Atlantic Water, our results indicate a particular importance of Atlantic Inflow for cod recruitment. The inter-annual temperature variations in this region and depth are around 2°C, therefore we doubt the direct influence of temperature on cod recruitment. The inflow of Atlantic Water is believed to transport zooplankton rich waters and thereby affect species composition of the zooplankton community in the North Sea [62], [63], [64], [31]. Our finding indirectly confirm that cod recruitment is rather influenced by the zooplankton transported with Atlantic Water [55] than by local temperature conditions. The question remains whether cod recruitment responds to the zooplankton composition of the Atlantic Water or to the strength (i.e. volume) of the inflow.

The significant correlation in the north-western part of the North Sea may indicate that the spawning stock component from that area is most important for the overall cod recruitment, i.e. recruits stemming from this area either prevail in numbers over other spawning/nursery areas or have the strongest inter-annual variability, which overrules the variability of the recruitment success elsewhere in the North Sea. Another possible explanation is that all stock components of North Sea cod are influenced by Atlantic inflow in a similar way. However, it is difficult to decide which explanation is plausible, without having a clear division of the North Sea cod stock into sub-stocks. The scales of larval transport [65], juvenile dispersal and adult movements [66], [67], [68], [69] suggest such division, but it is still under discussion [70], [71], [72]. There are numerous difficulties and uncertainties associated with the proposed sub-stocks’ boundaries (see e.g., discussion in [70]). For these reasons North Sea cod is currently assessed and managed as a single stock and only this assessment are used in our study. We suggest however that once such subdivisions are agreed, identification of main drivers of the sub-stocks’ variability may substantially contribute to our understanding of the overall stock dynamics of North Sea cod.

### European plaice and Common Sole

Recruitment of the European plaice showed negative correlation with temperature (Fig 4) in agreement with previous studies, which indicated negative correlations between temperature and year-class strength of plaice using data independent of stock assessments (e.g. pre-recruitment indices from research surveys, eggs abundances; [73], [74], [75]). Fox et al. [20] tested for correlation between temperature averaged within ICES areas and plaice recruitment, finding maximum correlations from February to June. Their results were lately confirmed by Brunel and Boucher [24] with longer time series. The season of the strongest correlation of Fox et al. [20] roughly agrees with our maximum correlation from April to June (Section ‘European plaice and temperature’). This time of the year corresponds to the larval and juvenile period of plaice, which is known to spawn in winter in the North Sea [28], [20].

Our results showed the strongest correlation between plaice recruitment and temperature in the German Bight, but no significant correlation in the Southern Bight or along the eastern British coast. The correlation was significant in the whole German Bight, overlapping with the plaice spawning areas there [76], whereas plaice nursery grounds are situated mainly inshore [77]. This may indicates the importance of German Bight as spawning/nursery area for the plaice in the North Sea, although the relative contribution of different spawning areas was shown to change over time [76].

Previous studies suggested diverse processes underlying the negative correlation between plaice recruitment and temperature. Several authors showed that the transport of the early-life stages of plaice from the spawning to the nursery areas (connectivity) is critical for the reproductive success, since the nursery areas of plaice are fixed in space [78], [76]. They argued that the interplay between circulation pattern and water temperature is decisive; specifically, in colder years slower development and growth rate of plaice eggs and larvae allows sufficient time to bridge the distance between plaice spawning and nursery areas before the onset of metamorphosis.

Negative correlations between fish recruitment and temperature are often associated with starvation mortality and match-mismatch mechanism during fish early-life stages ([79]). Starvation mortality may be caused by a decrease of suitable prey or by a temperature increase and higher metabolic costs. In agreement with Meyer et al. [80], AHOI temperature in the southern North Sea shows a weak positive trend in the period prior to 1980s and a strong increase afterwards. Furthermore, Rijnsdorp and van Leeuwen [81] showed a decrease of the growth of plaice juveniles in 1980s caused by eutrophication and beam trawling. We suggest that eutrophication could also affect food availability for plaice larvae in this period. The strong warming and changing feeding conditions might cause an onset of the food-limitation of the plaice larvae and the strengthening of the correlation between plaice recruitment and temperature since early 1980s (Fig 4B). A more detailed consideration of match-mismatch mechanisms for the survival of plaice larvae in the North Sea is needed to confirm or reject our suggestion.

Predation on the early-life stages was alternatively suggested to be a primary driver of the plaice reproductive successes in the North Sea. Based on findings of Pepin [82], Van der Land [83] and Fox et al. [20] assumed that the lower temperatures might cause a reduction of the predation pressure on plaice eggs and larvae, which would in turn enhance recruitment in cold years. However, their suggestion contradicts the study of Nash et al. [77], who claimed that the cumulative mortality during eggs and larval stages would increase with increasing stage duration at lower temperatures. Other sources of temperature-dependent mortality, such as embryonic malformation [84] or activity of pathogenic bacteria [85] were previously suggested to underlie the negative correlation between temperature and plaice recruitment. However, these sources of mortality are generally agreed to be rather small in comparison to starvation and predation mortality, as well as mortality linked to the connectivity [86], [87].

Sole and plaice have similar life history and feeding preferences in the North Sea [88]. Similar to plaice, connectivity has been shown to be important for the survival of young sole [89]. But there are some important differences between both flatfishes. Sole nursery areas are restricted to the southern North Sea (German and Southern Bights; [28]), whereas plaice nurseries are found in coastal waters throughout the North Sea (e.g. [76]). Another main difference is that plaice is a temperate species, which spawns in winter in the North Sea, whereas sole is a warm-water species, which normally spawns in late spring. This difference seems to be important for the feeding conditions and offspring growth in both species [88].

In general, there have been many fewer studies concerning the relations between sole recruitment and environmental conditions in comparison to plaice. Brunel and Boucher [24] found no significant correlation between temperature and sole recruitment at lag 0, similar to our results. Pécuchet et al. [22] found temperature being significantly correlated with sole pre-recruitment survival at lag 0. They used residuals from the Ricker stock-recruitment model fitted to the observed recruitment as the pre-recruitment survival index, therefore their results are difficult to directly compare with ours. We found correlations between R_s_/SSB_s_ and temperature at lag 1 and between R_s_ and temperature at lag 2. These lagged correlations point to a delayed response of sole recruitment to environmental changes. Such delayed response is normally ascribed to two main mechanisms [3]: 1) environmental factor influences species life history 2) environmental parameter influences trophic interactions of the species (availability of suitable prey and/or relevant predators). Determination of the exact mechanism behind the lagged response of sole recruitment to temperature changes will need further investigation.

### Atlantic Herring

Atlantic herring is one of the main commercial species in the North Sea and, due to its high biomass and a ‘wasp-waist’ trophic role, is believed to be one of the key agents of the trophic interactions (e.g. [16]). During almost 70 years of herring stock assessment, a strong collapse in 1970s and a stock recovery from 1990s were observed (Fig 3B). A substantial effort to understand the dynamic of this stock has been previously undertaken, mainly focusing on the dynamic of herring recruitment [29], [33], [90], [91], [92], [93]. We found no study about the influence of environmental changes on the biomass of adult herring in the North Sea.

Our analysis yielded a positive correlation between herring SSB_h_ and temperature at lag 0 (Fig 3) and negative correlation between temperature and R_h_/SSB_h_ at the same lag (not shown). The correlation analysis of herring stock variables showed that the variability of R_h_/SSB_h_ is mainly driven by the variability of SSB_h_ and not by the variability of R_h_ (see ‘Atlantic herring and temperature‘). Therefore we believe that the correlation between herring pre-recruitment survival index R_h_/SSB_h_ and temperature is due to the correlation between SSB_h_ and temperature and focus our discussion on the later relationship.

Although no correlation between herring spawning stock biomass and temperature has been previously reported, results of some early studies indirectly indicated such relation. Nash and Dickey-Collas [29] found a positive correlation between water temperature in the northern North Sea and herring young larvae abundance index, which was in turn highly correlated with the spawning stock biomass. The biomass of *Calanus finmachicus* (the main prey item of herring larvae) was shown to be negatively correlated with the temperature and therefore could not cause the positive correlation between larvae abundance and temperature [29]. Therefore, one can conclude that the positive correlation between larvae abundance and temperature is probably a consequence of a positive relation between spawning stock biomass and temperature. Supporting our results, Ottersen et al. [91] showed that including negative temperature effect into stock-recruitment model of the North Sea herring stock significantly improves explanatory power of the model for log-transformed pre-recruitment survival index, which is consistent with our finding of the negative correlation between temperature and R_h_/SSB_h_.

The strongest correlation between herring spawning stock biomass and temperature was found in the north-western North Sea (Fig 3A), where two of four spawning areas are located for autumn-spawning herring (Shetland and Buchan, see for example [94]). The region between Orkney and Shetland is known as an important feeding area of adult herring [95], [96], [97], which primary feed on zooplankton (*Calanus*, *Temora*, *Oikopleura*, *Amphipoda*), although fish eggs, larvae and small clupeid fishes can comprise significant portion of its diet [98], [99], [100]. Tight links between herring and zooplankton distribution as well as between herring migration and zooplankton seasonal cycles have been previously shown [101], [63].

As we mentioned above for cod, temperature in the north-western North Sea is driven by a combination of local atmospheric conditions and remote temperature changes in the North-Atlantic. The strongest correlation between herring spawning stock and temperature was found at 60 m (Fig 3), i.e. in a layer relatively isolated from the atmosphere. Similar to the case of cod, this indicates that changes of herring spawning stock biomass are more strongly influenced by changes of Atlantic inflow than by the variability of the air temperature. Several mechanisms might come into play here. Enhanced temperature may directly influence the growth rate of herring and thereby cause enhanced stock biomass in warm years. However, as we mentioned, the temperature amplitudes in this region are around 2°C on the inter-annual scale, therefore we rather suggest that temperature is a proxy for other mechanisms driving variability of herring spawning stock. One likely candidate might be changes in the zooplankton composition of the Atlantic inflow and, hence, the quantity of food for adult herring. A visual comparison of herring SSB_h_ with the time series of the zooplankton species in the North Sea [62], [64] did not yield clear match. We hope that a detailed and accurate comparison of existing time series could shed more light on the mechanisms driving the variability of herring stock in the North Sea.

In order to understand whether herring spawning stock biomass is primarily driven by fishery or by environmental changes, we calculated cross-correlation between herring fishing mortality F_h_ and SSB_h_. We found the strongest correlation of -0.71 between the time series when the F_h_ was lagged by 2 years. Therefore, the effect of fishing on SSB_h_ seems roughly comparable to the effect of climatic changes. Additional analysis is needed to determine which factor primarily drives the variability of the herring stock in the North Sea.

### European Sprat

Among commercially important fish species in the North Sea, European sprat is perhaps the least studied in terms of the influence of environmental conditions on its stock dynamics. From an ecological perspective, sprat is an abundant small pelagic fish species and play an important role in the trophic dynamics of marine ecosystems [102], [103]. The sprat stock in the North Sea underwent several declines during the last four decades, the most dramatically at the end of 1970s (Fig 6B). Our results suggest that these declines are not caused by sprat fishery alone, because no significant correlation between sprat fishing mortality and spawning stock biomass was found (Section ‘European Sprat and Salinity’). As discussed within ICES, 1990 [104], sprat catches decreased apparently at the same time period over a large area and, therefore, were probably caused by changing climatic conditions. The abundance and recruitment of the Baltic Sea sprat has been shown to be linked to salinity changes [105], [106]. To our knowledge, our study illustrates for the first time a correlation between inter-annual salinity variability and sprat stock dynamics in the North Sea, although the relationships between salinity and sprat distribution has been suggested previously [107], [108].

Our analyses revealed a positive correlation between salinity and two sprat stock variables: recruitment and spawning stock biomass. Both stock variables are highly correlated with each other (P(τ = 0) = 0.72). Since sprat is a short-lived species, the 0-group fishes are believed to comprise a significant proportion of the total biomass [13]. Therefore it is unclear whether sprat SSB_sp_ drives the variability of sprat R_sp_ in the North Sea or R_sp_ contributes to the variability of SSB_sp_. A significant correlation between salinity and sprat SSB_sp_ was found in the western North Sea (Fig 6A). This area overlaps with known spawning areas of sprat [104], [109], [110]. The region of significant correlation with sprat R_sp_ covers the whole central and southern North Sea (Fig 7A), coinciding with main nursery area of the North Sea sprat [111], [112].

Inter-annual variability of salinity in the North Sea is mainly driven by the inflow of highly saline Atlantic water in the north [61], [113], [114], [115] and river discharge from continental Europe in the south [116], [117]. A thorough study is needed to identify the primary salinity driver in the central and south-western North Sea, where the strong correlation with sprat stock variables was found. However, it seems unlikely that salinity affects the sprat distribution or growth directly, because sprat is known to tolerate a wide range of salinities and inhabits waters from the Mediterranean to the Baltic Seas [25], [118]. We rather suggest that salinity is a tracer of other biotic or abiotic conditions that seem to be important for sprat. European sprat is strictly planktivorous during its whole life cycle [98], [99], [119], and therefore should be sensitive to changes in the amount/composition of zooplankton in the North Sea. As we already mentioned, Atlantic water inflow is known to affect zooplankton composition in the North Sea [31], [62], [64]. Rivers discharge nutrient rich waters and strongly affect water stratification in the North Sea [117], both are important factors influencing primary and secondary productivity. A more detailed investigation of this bottom-up control of the sprat variability is needed to discover exact mechanism behind the correlations found in our study.

### Management applications

As in many other parts of the world, commercially important fish stocks in the North Sea are managed by annual quotas, which are currently based on the Maximum Sustainable Yield (MSY) concept [120]. To estimate MSY, and the associated fishing mortality and stock biomass, many aspects of the stock's biology, including the stock-recruitment relationships (SRRs) have to be taken into account and used for the projections of the future stock development. However, SRR models typically have low predictive power given that changes in the productivity of stocks can be partially driven by other factors than changes in SSB (e.g., [21], [121]). The incorporation of environmental covariates in SRR models was shown to improve significantly their fit to historical data (e.g. North Sea cod, [122], [123]), and such environmental influence may be incorporated into future predictions of fish stocks as well. Fisheries management needs to be responsive to ensure a sustainable exploitation under different hydrographic regimes.

Our study supports the notion of profound influence of climate forcing on stock dynamics of 5 commercially important fishes in the North Sea. Some of the correlations found in this study have been shown previously, but we were able to increase the explanatory power of the environmental covariates by identifying regions of strongest correlation. As one of many possible approaches for including such relationships in management, we propose that time series of temperature and salinity in the regions of maximum correlations may be further used in SRR models that take into account environmental effects in different ways (e.g. [124], [125]). The best model for each fish species can be chosen based on the Akaike Information Criterion (AIC, [126]) or cross-validation and can provide insight into the possible influences of environmental factors to fish stocks. For example, environmental factors may control the stock through mortality rates, or limit it by altering the carrying capacity, etc. (see [124]). Such an approach is (at least partially) mechanistic and should be most effective for management [127].

As an example, we show how this approach might be applied to North Sea cod. We followed the methodology of Levi et al. [128], whereby various types of SRRs were fitted to historical data, some of which were modified to incorporate differing types of environmental effects (e.g. 'limiting', 'controlling' and 'masking' effects). Fitting was performed using a non-linear least squares approach with log-transformed versions of each model (Table 3). The temperature time series that best correlated to recruitment dynamics (Fig 2B) was used as the environmental covariate. Both AIC and cross-validation statistics showed significant improvements in fit for models that incorporated environmental effects, with a Cushing-type model incorporating 'environmental masking' as the best overall (Table 3).

**Table 3:** Summary statistics for fitted SRR model to historic data on spawning stock biomass (SSB) and recruitment (R) of the North Sea cod used in this study.

SRR type	Env. effect	Formula	Formula (log-transformed)	AIC	R^2^	RMSE*	MdAPE*
Cushing	none	R = αSSB^γ^	log(R) = log(α) + γlog(SSB)	96.2	0.505	45.10	0.608
	Controlling	R = αSSB^γ^e^c*E*^	log(R) = log(α) + γlog(SSB) + c*E*	(3) 76.5	(3) 0.676	(3) 35.65	(2) 0.508
	Masking	R = αSSB^γ+c*E*^	log(R) = log(α) + γlog(SSB) + clog(SSB)*E*	(1) 76.3	(1) 0.678	(1) 35.36	(1) 0.506
Beverton-Holt	none	R = SSB/(b + aSSB)	log(R) = log(SSB/(b + SSB))	97.4	0.496	45.06	0.620
	Controlling	R = SSBe^c*E*^/(b + aSSB)	log(R) = log(SSB/(b + aSSB) + c*E*	76.6	0.676	35.81	0.509
	Masking	R = SSB/(be^c*E*^ + aSSB)	log(R) = log(SSB/(be^c*E*^ + aSSB)	78.3	0.665	37.36	0.512
	Limiting	R = SSB/(b + ae^c*E*^SSB)	log(R) = log(SSB/(b + aSSBe^c*E*^)	80.6	0.652	35.67	0.525
Ricker	none	R = αSSBe^−βSSB^	log(R) = log(α) + log(SSB) – βSSB	97.3	0.496	45.08	0.620
	Controlling	R = αSSBe^−βSSB^e^c*E*^	log(R) = log(α) + log(SSB) – βSSB + c*E*	(2) 76.5	(2) 0.677	35.83	(3) 0.508
	Masking	R = αSSBe^−βSSB(1+c*E*)^	log(R) = log(α) + log(SSB) – βSSB + cSSB*E*	78.5	0.664	(2) 35.45	0.522

*E* represents the environmental variable (in this particular case, temperature anomalies at 60.0°N, 4.4°W at 75 m depth; Fig 2), a, b, c, α, β, γ are fitted coefficients. AIC is Akaike information criterion, R^2^ is R-squared (log-transformed recruitment); RMSE is root-mean-square error (log-transformed recruitment) and MdAPE is median-absolute-percent error (recruitment).

* indicates statistics calculated from a 4-fold cross-validation procedure (50 permutations). Numbers in parenthesis indicate rankings of the top three performing models for each statistic.

**Fig 8:**
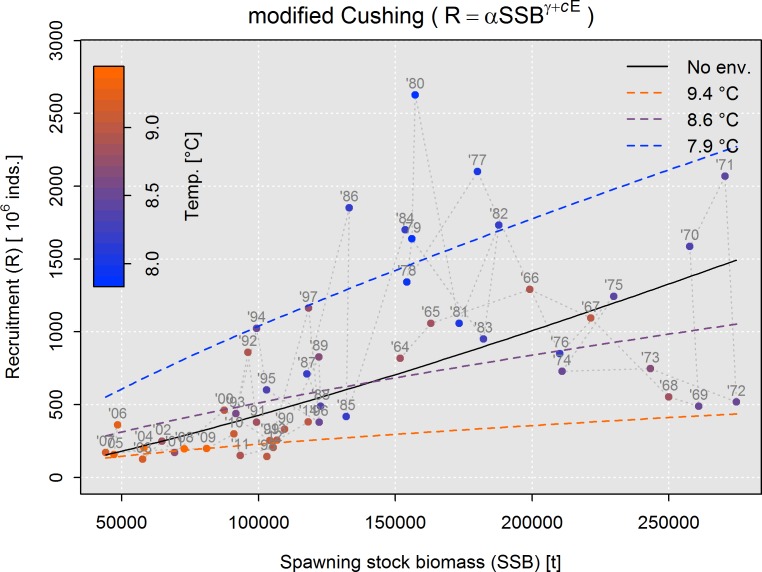
Fitted stock-recruitment relationship for North Sea cod (modified Cushing model with 'environmental masking'). Predicted recruitment for three temperatures during JFM is shown (colored dashed curves), as well as the prediction of the non-environmentally-mediated Cushing model for reference (solid black curve). Points show specific stock-recruitment observations. The colors indicate the temperature in JFM and the corresponding recruitment year is in grey text.

Fig 8 shows the prediction of the best fitting model (modified Cushing model with 'environmental masking') against historical data and illustrates the significantly lower recruitment associated with warmer temperatures in recent years. The model terms for spawning stock (log(*SSB*)) and its interaction with temperature (log(*SSB*)*E*) were both evaluated via an F-test to be highly significant (p < 0.001). Without environmental effects (solid black curve in Fig 8), the model is of much poorer fit and the spawning stock biomass term is slightly positive (*γ* = 1.24). Such a convex relationship may imply a depensatory mechanism at low spawning stock levels, but is unlikely to be representative at higher spawning stock levels [129].

Once having a model linking environmental changes to the fish stock, prognostics relating the environmental variables can be used in management. There are, however, some reasonable critiques of the incorporation of environmental covariates in predictive SRR models for management purposes. These critiques seem to prevent tactical management from including environmental drivers into stock predictions, as it was highlighted by a recent review which found that physical and biological ecosystem drivers were only implemented in the management of 24 out of more than 1200 marine fish stocks worldwide [130]. The first criticism concerns the lack of mechanistic understanding of the links between environmental factors and stock parameters and possible breaking down of correlations if circumstances change. Such breaks have been demonstrated previously for various stocks [131] and have serious consequences if environmental drivers are included into management plans (see examples in Skern-Mauritzen et al. [130]). However, several decades of research focusing on the mechanistic understanding of the correlative relationships have proven extremely difficult to discover precise mechanisms. Furthermore, extrapolating beyond historically observed climatic regimes is challenging independently of the detail of mechanistic understanding. As we mentioned, our approach is based on modified models incorporating different types of environmental effects related to possible mechanisms and is partially mechanistic. Moreover, we agree with Planque et al. [123], who suggested that management can still use the relationships between stock and environmental variables without fully understanding of the underlying mechanisms. The correlations found in this study are statistically robust and most of them are valid over a long time period. A regular reviewing of such correlations with newly available data would be a possible way to handle the uncertainty about the correlative relationships.

The second criticism concerns the unreliability of predictions of the environmental factors, as it was argued by Walters and Collie [132] and Planque et al. [123]. Reliable predictions have been mainly achieved at seasonal time scale or at the scale of several decades (e.g. [133], [134], [135]). Some of the long-term predictions have been recently used in fishery related studies to predict trends over medium to long-term scales, such as decadal oscillations or climate change shifts (e.g. [136]). However, reliable forecasts at the time scales most relevant for management, i.e. short (1–2 years) and medium (5–10 years) term, are still challenging [132]. We believe that the predictability of the North Sea hydrography at short time scales can be improved in some cases, as it has been shown by Planque et al. [123]. We suggest that the short-term hydrography prognostics can be achieved with statistical models, for instance, autoregressive models or linear models involving relations to climatic indices and teleconnections. One example would be the salinity of the southern and central North Sea, which was shown here to be a good environmental covariate of sprat SSB and recruitment. Schott [116] and Heyen and Dippner [117] showed that the salinity in the southern North Sea is correlated to river discharge with 1 year lag. Therefore, there is a potential to predict the sprat stock’s dynamics with at least 1 year in advance based on the observations of river discharge. Another candidate is the temperature of the Atlantic water in the north-western North Sea, which correlates with the recruitment of the North Sea cod at 1 year lag. The inter-annual variability of the temperature in the northern North Sea may be predicted with at least one year in advance using temperature observations in the Rockall Trough in the eastern North Atlantic. Such lagged relations have been shown previously for salinity [61]. Of course, detailed analyses are needed to estimate the explanatory power and uncertainties of such forecasts of the future trajectories of fish stocks in the North Sea.
